# Investigating Sequence Variations in CNTNAP2 and SETBP1 Genes in Language Disorders

**DOI:** 10.1192/j.eurpsy.2025.1145

**Published:** 2025-08-26

**Authors:** B. Turan, E. Göktaş, N. Uzun, A. T. Hıra Selen, A. G. Zamani, M. S. Yıldırım

**Affiliations:** 1 Medical Genetics; 2 Child and Adolescent Psychiatry, Necmettin Erbakan University, Konya, Türkiye

## Abstract

**Introduction:**

Language Disorder, a prevalent developmental disorder, impedes children’s communication skills, with genetic and environmental factors playing pivotal roles in its pathomechanism.

**Objectives:**

This study aims to investigate the involvement of sequence variations in *SETBP1* and *CNTNAP2* genes, along with environmental variables, in Language Disorder’s etiology.

**Methods:**

Between September 2022 and March 2023, thirty children aged 2-7 diagnosed with language disorders according to DSM-5 criteria, and evaluated using the Ankara Developmental Screening Inventory, were studied to identify genetic and environmental factors contributing to etiology.Thirty healthy children with similar age were included as a control group. DNA samples isolated from peripheral blood of both groups were analyzed for *SETBP1* and *CNTNAP2* genes using next-generation sequencing (custom design panel). The frequencies and clinical significance of the identified variants was evaluated, and variant verification and segregation analyses were performed by Sanger sequencing. The obtained data were compared using appropriate statistical methods.

**Results:**

Language Disorder showed a male-dominant distribution. The *SETBP1* rs11082414-CC genotype frequency was significantly higher in patients (p=0.024), and two rare variants (CNTNAP2: c.973C>G:p.P325A; *CNTNAP2:* c.2236G>A:p.D746N) were exclusive to cases. In silico analyses yielded conflicting results for rare variants, inherited paternally from unaffected parents. Among non-genetic factors, patients had higher birth weights (p=0.043) and shorter lactation durations (p=0.044).

**Conclusions:**

Homozygosity for *SETBP1* rs11082414 polymorphic variant increases Language Disorder susceptibility. This study underscores the genetic dimension of Language Disorder, urging physicians’ awareness and early intervention strategies to mitigate its impact.

**Disclosure of Interest:**

None Declared

